# Association of Single Nucleotide Polymorphisms in the Cyclooxygenase-2 (COX-2) Gene with Periodontal Disease—A Systematic Review with Meta-Analysis and Implications for Personalized Dentistry

**DOI:** 10.3390/jpm15080351

**Published:** 2025-08-03

**Authors:** Vasiliki Savva, Ioannis Fragkioudakis, Dimitra Sakellari

**Affiliations:** Department of Preventive Dentistry, Periodontology and Implant Biology, Dental School, Faculty of Health Sciences, Aristotle University of Thessaloniki, Thessaloniki 54124, Greece; vsavvaa@dent.auth.gr (V.S.); ifragkio@dent.auth.gr (I.F.)

**Keywords:** periodontal disease, cyclooxygenase-2 (COX-2), COX-2 single-nucleotide gene polymorphism, genetic susceptibility

## Abstract

**Background:** Genetic polymorphisms in the cyclooxygenase-2 (COX-2) gene may contribute to individual susceptibility to periodontal disease. A meta-analysis assessed the association between three COX-2 single-nucleotide polymorphisms (SNPs) namely, −765 G/C (rs20417), −1195 G/A (rs689466), and 8473 T/C (rs5275), and the risk of CP. **Methods:** Following the PRISMA 2020 guidelines, we conducted a comprehensive search of five electronic databases and additional sources. The eligible studies were observational (case–control or cohort) with genotypic data comparing individuals with periodontal disease and periodontally healthy controls. Methodological quality was assessed using the Newcastle–Ottawa Scale (NOS), and the certainty of evidence was evaluated via the GRADE framework. Pooled odds ratios (ORs) with 95% confidence intervals (CIs) were calculated under dominant genetic models. **Results:** Seven studies (n = 1467 participants) met the inclusion criteria. No eligible studies evaluated the 8473 T/C SNP. The meta-analysis of the −765 G/C variant revealed a significant association with periodontal disease (OR = 1.61; 95% CI: 1.12–2.32, *p* = 0.03; I^2^ = 0%). For the −1195 G/A variant, the pooled OR was 1.86 (95% CI: 1.00–3.43, *p* = 0.05; I^2^ = 35%), suggesting a borderline significant association. The certainty of evidence was graded as moderate for −765 G/C and low for −1195 G/A. **Conclusions:** The COX-2 −765 G/C polymorphism is significantly associated with increased CP risk, while the −1195 G/A variant shows a potential, though less certain, link. Larger, high-quality studies using standardized classifications are needed to confirm these associations.

## 1. Introduction

Periodontal disease is a chronic, multifactorial inflammatory disease that affects the supporting structures of the teeth, leading to progressive destruction of the periodontal ligament, alveolar bone loss, and ultimately tooth loss [1]. It is one of the most prevalent oral diseases worldwide, affecting approximately 20% to 50% of the global population with varying degrees of severity [2]. The primary etiological factor of periodontal disease is the microbial biofilm that forms in the gingival sulcus. However, disease progression is not solely due to the presence of bacteria, but also to the host immune-inflammatory response, which eventually leads to tissue damage [3,4].

In addition, several systemic and environmental modifiers, including smoking, diabetes, and stress, as well as genetic predisposition, are known to influence susceptibility to periodontal breakdown [4]. Several studies have pointed toward elevated levels of inflammatory biomarkers and the presence of specific cytokine gene polymorphisms as contributing to inter-individual variability in disease risk [5]. Specifically, polymorphisms in cytokine-encoding genes, such as those of interleukin-1 (IL-1), have been associated with differential inflammatory responses to periodontal pathogens. Numerous studies have shown that single-nucleotide polymorphisms (SNPs) in the *IL1A* (−889 C/T; rs1800587) and *IL1B* (+3953 C/T; rs1143634) genes can contribute to heightened expression of IL-1α and IL-1β, thereby exacerbating local tissue inflammation and destruction [6]. The presence of the so-called composite PST+ genotype, characterized by carriage of the T allele at both loci, has been associated with an increased risk of severe periodontitis, with odds ratios ranging from 1.5 to 7.0, depending on ethnicity and environmental cofactors [6]. Conversely, homozygosity for the C allele at both positions has been suggested to confer a degree of protection against disease progression. These findings underscore the multifactorial nature of periodontitis and support the integration of genetic risk profiling in the broader framework of periodontal risk assessment.

Cyclooxygenase (COX), a critical enzyme in the arachidonic acid pathway, catalyzes the production of prostanoids—primarily prostaglandins (PGs)—which are key mediators of inflammation. COX exists in two isoforms: COX-1, which is constitutively expressed in most tissues, and COX-2, which is inducible and upregulated during inflammatory processes [7,8]. COX-2 plays a pivotal role in the biosynthesis of prostaglandin E_2_ (PGE_2_), which contributes to vasodilation, increased vascular permeability, soft tissue degradation, and bone resorption in periodontal lesions [9,10].

Numerous cell types in periodontal tissues—including fibroblasts, osteoblasts, epithelial cells, endothelial cells, polymorphonuclear leukocytes, monocytes, and macrophages—are stimulated by inflammatory mediators such as interleukin-1β, tumor necrosis factor-α, and lipopolysaccharide from Gram-negative bacteria to upregulate COX-2 expression [11]. This results in increased local PGE_2_ concentrations, which, in concert with cytokines and matrix metalloproteinases (MMPs), drive the degradation of the extracellular matrix and alveolar bone.

Genetic variations in the COX-2 gene may influence its transcriptional regulation, mRNA stability, or enzymatic function, thereby modulating the intensity of inflammatory responses. Several single-nucleotide polymorphisms (SNPs) have been identified in the COX-2 gene, including rs20417 (−765G/C), rs689466 (−1195G/A), and rs5275 (8473T/C), among others [12,13]. These polymorphisms have been investigated for their potential roles in susceptibility to periodontal disease, with varying and often conflicting results across populations and study designs.

To date, no clear consensus has been reached regarding the relationship between COX-2 gene polymorphisms and chronic periodontitis. While individual case–control studies may lack statistical power due to small sample sizes or population heterogeneity, meta-analysis allows for the aggregation of findings across multiple studies to yield more robust and generalizable conclusions. The potential application of COX-2 polymorphisms in personalized periodontal care is a growing area of clinical interest. As part of a precision medicine approach, identifying genetic susceptibility markers may enable early diagnosis, risk stratification, and targeted anti-inflammatory interventions. This is particularly relevant for populations with a high prevalence of periodontal disease or known exposure to environmental modifiers such as smoking. Therefore, the present meta-analysis aimed to evaluate the association between two well-characterized COX-2 polymorphisms, −765G/C (rs20417) and −1195G/A (rs689466), and periodontal disease, synthesizing the available evidence from multiple populations [14]. Until 2017, periodontal diseases were classified according to the 1999 International Workshop for a Classification of Periodontal Diseases and Conditions, which distinguished between chronic and aggressive periodontitis based on clinical, epidemiological, and pathophysiological criteria. The studies included in this meta-analysis were published before the adoption of the revised classification system of 2018, which replaced the chronic/aggressive distinction with a staging and grading framework [15]. Therefore, the present review refers to periodontal diagnoses as reported initially by the authors, in line with the 1999 classification, to maintain consistency and avoid retrospective misclassification. The term “periodontal disease” is used throughout the review to reflect the terminology employed in the original publications.

The purpose of this systematic review and meta-analysis is to comprehensively assess the association between COX-2 gene polymorphisms and susceptibility to periodontal disease, and to explore how this association varies across populations and disease classifications. This work aims to contribute to the growing field of personalized dentistry by identifying genetic markers that may support risk stratification and individualized prevention strategies.

## 2. Materials and Methods

This systematic review and meta-analysis were conducted in accordance with the PRISMA 2020 (Preferred Reporting Items for Systematic Reviews and Meta-Analyses) guidelines. A detailed protocol was established in advance to ensure transparency and methodological consistency across all stages, from literature search and study selection to data extraction, quality appraisal, and statistical synthesis. 

The protocol was registered in the Prospective Register of Systematic Reviews (PROSPERO; registration number: CRD420251008939) on 26 March 2025 and is accessible at: https://www.crd.york.ac.uk/PROSPERO/view/CRD420251008939 (accessed on 29 July 2025).

### 2.1. Eligibility Criteria

Eligibility was defined using the PECO framework:

Population (P): Individuals diagnosed with periodontal disease, including chronic or aggressive forms, as defined by either the 1999 American Academy of Periodontology (AAP) classification system or the updated 2017 AAP/European Federation of Periodontology (EFP) classification. Both diagnostic frameworks were accepted due to their relevance in different periods and their wide use in epidemiological and genetic studies.

Exposure (E): the presence of cyclooxygenase-2 (COX-2) gene polymorphisms, specifically −1195 G/A (rs689466), −765 G/C (rs20417), and 8473 T/C (rs5275), which have been proposed as potential genetic susceptibility markers for periodontal disease.

Comparison (C): individuals with periodontal health, defined as the absence of clinical attachment loss, probing pocket depth greater than 3 mm, and radiographic evidence of alveolar bone loss.

Outcome (O): the presence of periodontal disease, assessed about the COX-2 gene polymorphisms through genotypic distribution, odds ratios (ORs), 95% confidence intervals (CIs), and corresponding *p*-values.

Only observational studies with case–control or cohort design were considered eligible. Studies were excluded if they met any of the following criteria:

Did not report genetic data relevant to COX-2 polymorphisms.

Lacked a periodontally healthy control group.

Failed to examine at least one of the three target polymorphisms.

Were non-human or in vitro studies, reviews, editorials, or conference abstracts.

Included fewer than 50 participants per group (unless part of a larger pooled dataset).

As part of quality control, the Hardy–Weinberg Equilibrium (HWE) was assessed for the control group in each study. Studies showing deviation from the HWE were excluded in sensitivity analyses.

### 2.2. Information Sources and Search Strategy

A comprehensive literature search was performed across five major electronic databases: PubMed (MEDLINE), Web of Science, Scopus, EMBASE, and the Cochrane Library. To minimize the risk of publication bias and identify the relevant gray literature, supplementary searches were conducted via Google Scholar. Furthermore, the reference lists of all included articles were manually screened to retrieve any additional eligible studies. The search strategy combined Medical Subject Headings (MeSH) and free-text keywords related to COX-2 gene polymorphisms and chronic periodontitis, as well as periodontal disease, using Boolean operators (AND, OR) for logical structuring. No time restrictions were imposed on publication date, and only full-text studies published in English were included (Appendix A).

### 2.3. Study Selection

Two independent reviewers (V.S. and I.F.) screened the titles and abstracts of all retrieved articles for relevance. Full texts of potentially eligible studies were then examined against the predefined inclusion and exclusion criteria. In cases of disagreement, consensus was reached through discussion, with the involvement of a third reviewer (D.S.) when necessary. The entire selection process was documented using a PRISMA flow diagram, which detailed the number of records identified, screened, excluded, and ultimately included in the final analysis.

### 2.4. Data Extraction

Data were extracted independently by the two reviewers using a standardized and piloted data collection form. The following variables were recorded: authorship, year of publication, country of origin, participant ethnicity, age, gender, smoking status, genotype and allele frequencies for each COX-2 polymorphism, statistical measures (including ORs, 95% CIs, and *p*-values), HWE status in the control group, and whether adjustments for confounding factors such as smoking or diabetes had been made. Discrepancies in the extracted data were resolved through joint review and discussion.

### 2.5. Quality Assessment

The methodological quality of the included studies was evaluated using the Newcastle–Ottawa Scale (NOS), a standardized tool for assessing non-randomized studies. This scale examines three domains:

(1) Selection of study groups;
(2) Comparability between cases and controls;
(3) Ascertainment of exposure.

Each study was assigned a score out of a maximum of 9 points. Studies that achieved a score of 7 or higher were classified as high quality, while those scoring 6 or below were considered of lower quality. Lower-quality studies were not excluded but were incorporated into sensitivity analyses to evaluate their potential impact on the overall results.

### 2.6. Statistical Analysis

Quantitative synthesis was conducted using Review Manager (RevMan 5.4) and STATA software. The strength of association between COX-2 polymorphisms and periodontal disease was estimated using odds ratios with 95% confidence intervals. Analyses were performed under multiple genetic models, including allelic (e.g., G versus A for −1195 G/A), dominant (e.g., GG + GA versus AA), and recessive (e.g., GG versus GA + AA) models. Heterogeneity among studies was assessed using the I^2^ statistic, with values below 25% considered low, between 25% and 50% moderate, and above 50% substantial. Cochran’s Q test was also applied to evaluate the statistical significance of heterogeneity. Depending on the degree of heterogeneity, either a fixed-effects or random-effects model was used, with the latter being preferred in the presence of high heterogeneity.

### 2.7. Assessment of Publication Bias

To evaluate the risk of publication bias, funnel plots were generated for visual inspection of asymmetry, and Egger’s regression test was applied to detect small-study effects. In cases where evidence of bias was detected, the trim-and-fill method was used to estimate the number of potentially missing studies and adjust the meta-analytic results accordingly.

### 2.8. Ethical Considerations

As this review relied exclusively on secondary analysis of data extracted from previously published studies, no ethical approval was required. Nevertheless, all procedures adhered to the ethical principles relevant to systematic reviews, including accurate reporting, transparency, and integrity in data synthesis and interpretation.

### 2.9. Certainty of Evidence

The overall certainty of evidence for each outcome was assessed using the GRADE (Grading of Recommendations Assessment, Development and Evaluation) approach, taking into account factors such as risk of bias, inconsistency, indirectness, imprecision, and publication bias.

## 3. Results

### 3.1. PRISMA Flow and Study Selection Results

The initial search strategy identified a total of 1124 records across five electronic databases, including PubMed (MEDLINE), Scopus, Web of Science, EMBASE, and the Cochrane Library. An additional 36 records were retrieved from supplementary sources, including Google Scholar and manual searches of reference lists, yielding a combined total of 1160 records.

After removing 218 duplicate entries, 942 unique records remained for title and abstract screening. Based on predefined eligibility criteria, 865 records were excluded during this initial screening phase due to irrelevance, lack of genetic data, or study type (e.g., reviews, in vitro studies, or animal experiments).

The full texts of 77 articles were retrieved and assessed for eligibility. Following a detailed evaluation, 70 studies were excluded. The most frequent reasons for exclusion included the absence of a healthy control group (n = 19), non-assessment of any three COX-2 polymorphisms of interest (n = 17), incomplete or non-extractable genotypic data (n = 18), and insufficient sample size (n = 16).

**Figure 1:**
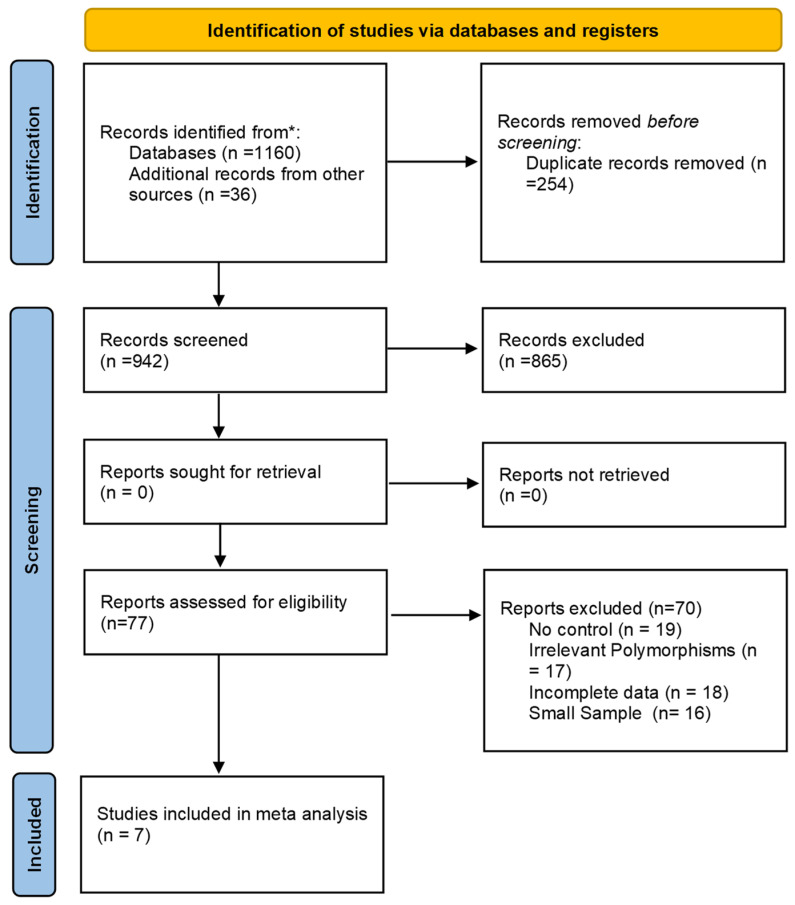
PRISMA flow diagram data summary. * Databases searched included PubMed (MEDLINE), Scopus, Web of Science, EMBASE, and the Cochrane Library. Additional records were retrieved from Google Scholar and manual reference list searches.

Ultimately, seven studies met all inclusion criteria and were included in the qualitative synthesis. These studies provided sufficient data to be incorporated into quantitative meta-analysis. The complete selection process is illustrated in the PRISMA flow diagram (Figure 1).

### 3.2. Study Characteristics

Seven studies were included in the final analysis, published between 2009 and 2023, and conducted across various geographical regions, including Asia and the Middle East. The study populations represented diverse ethnic backgrounds such as Chinese, Indian, Iraqi, and other Asian groups. All included studies that employed a case–control design to examine the association between COX-2 gene polymorphisms—specifically −765 G/C, −1195 G/A, and 8473 T/C—and the presence of periodontal disease.

Sample sizes across studies ranged from 100 to over 400 participants, with a balanced distribution of cases and controls in most studies. Mean participant age, gender distribution, and smoking status were reported variably, although most studies accounted for these factors during recruitment or analysis. All three COX-2 polymorphisms were not universally evaluated across studies; some focused exclusively on one or two variants.

The key characteristics of the included studies are summarized in Table 1.

### 3.3. Risk of Bias Within Studies

The methodological quality of the seven studies included in this meta-analysis was evaluated using the Newcastle–Ottawa Scale (NOS). This tool assesses studies across three domains: selection (up to 4 points), comparability (up to 2 points), and exposure (up to 3 points), giving a total score ranging from 0 to 9.

Only two studies, Li et al., and Loo et al. [18,19], achieved scores of 7, indicating high methodological quality, primarily due to appropriate control selection, valid outcome ascertainment, and adequate control for confounding variables (Table 2). Xie et al., [20] received a score of 6 and was classified as moderate quality, lacking points in control-selection domains.

The remaining four studies—Prakash et al. [7], Dahash et al. [14], Daing et al. [16], and Dienha et al. [17]—were rated as low quality, with total scores ranging from 4 to 5. Most of these studies lost points due to non-representative case selection, the absence of detailed control group selection criteria, and limited control for confounding factors such as smoking status or systemic health.

Overall, only two out of seven studies (29%) were classified as high quality (NOS ≥ 7), while one was of moderate quality, and four were of low quality (≤5 points). These assessments were used to inform subgroup and sensitivity analyses, evaluating whether methodological quality influenced the pooled effect estimates.

### 3.4. GRADE Assessment of Certainty of Evidence

The certainty of evidence, assessed using the GRADE framework, was rated as moderate for the association between the COX-2-765 G/C polymorphism and periodontal disease, and low for the −1195 G/A polymorphism, primarily due to imprecision, methodological limitations, and potential publication bias. These ratings reflect varying degrees of confidence in the observed associations and their applicability across populations (Appendix A).

### 3.5. Results of Individual Studies

Seven studies evaluated the association between COX-2 gene polymorphisms and periodontal disease. For the −765 G/C polymorphism, three studies reported dominant model comparisons (GC + CC vs. GG) and found varied strengths of association, with Li [18] and Loo [19] showing statistically significant increased risk, while Prakash et al. [7] reported a non-significant association. For the −1195 G/A polymorphism, four studies provided dominant model comparisons (GA+AA vs. GG), with Xie et al. [20] and Dienha et al. [17] showing significant associations, while Prakash et al. [7] and Daing et al. [16] reported non-significant findings. No eligible studies evaluating the -8473 T/C variant met the inclusion criteria. Specifically, no articles reporting on genotype distributions or associations between the—8473 T/C SNP and periodontitis in case–control or cohort designs were identified. Therefore, this SNP was not included in the meta-analysis due to insufficient available data.

Table 3 summarizes the genotypic distributions and the odds ratios (ORs) with 95% confidence intervals (CIs) and *p*-values from each individual study.

### 3.6. Synthesis of Results—Meta-Analysis

COX-2 −765 G/C Polymorphism

**Figure 2:**
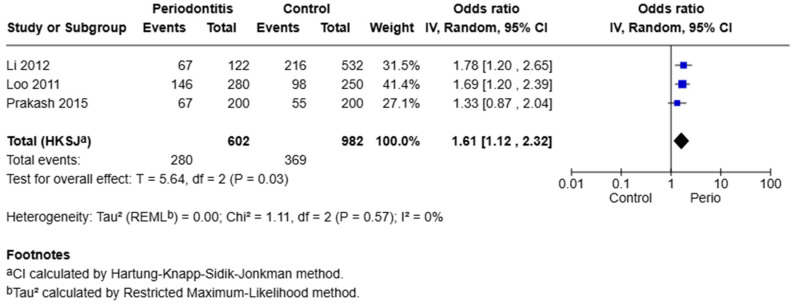
Forest plot for the association of the COX-2 −765 G/C polymorphism with periodontitis under the dominant model (GC + CC vs. GG). Data were derived from the studies of Li et al. [18], Loo et al. [19], and Prakash et al. [7]. The size of each square reflects the weight of the study, and the horizontal lines represent 95% confidence intervals. The pooled estimate was calculated using a random-effects model.COX-2 −1195 G/A Polymorphism.

A meta-analysis of three studies evaluating the −765 G/C polymorphism under the dominant model (GC + CC vs. GG) revealed a significant association with periodontal disease. The pooled odds ratio was 1.61, with a 95% confidence interval of 1.12 to 2.32 (*p* = 0.03). The heterogeneity among these studies was negligible (I^2^ = 0%, Q = 1.11, *p* = 0.57). This indicates that individuals carrying at least one C allele (GC or CC genotype) have 61% higher odds of developing periodontal disease compared to individuals with the GG genotype (Figure 2).

**Figure 3:**
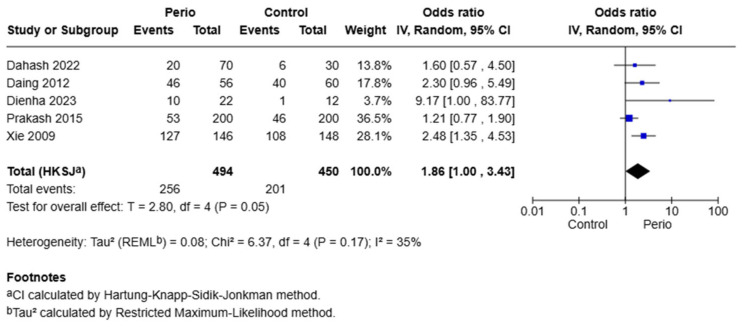
Forest plot for the association of the COX-2 −1195 G/A polymorphism with periodontitis under the dominant model (GA + AA vs. GG). Data were derived from the studies of Dahash et al. [14], Daing et al. [16], Dienha et al. [17], Xie et al. [20], and Prakash et al. [7]. The size of each square reflects the weight of the study, and the horizontal lines represent 95% confidence intervals. The pooled estimate was calculated using a random-effects model.

Five studies were included under the dominant model (GA + AA vs. GG) for the −1195 G/A polymorphism. The pooled odds ratio was 1.86 (95% CI: 1.00–3.43, *p* = 0.05), indicating a borderline significant association. Heterogeneity was moderate (I^2^ = 35%, Q = 6.37, *p* = 0.17), supporting the use of a random-effects model. The borderline significant OR of 1.86 suggests that carriers of the A allele may have an 86% increase in the odds of developing the disease. However, this finding should be interpreted with caution due to moderate heterogeneity and the wide confidence interval (Figure 3).

### 3.7. Publication Bias

**Figure 4:**
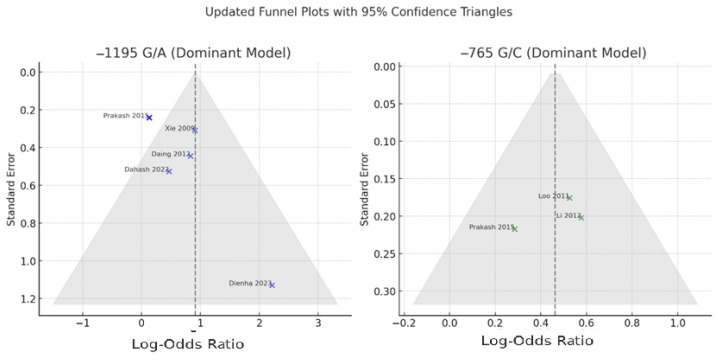
The final funnel plots for both polymorphisms under the dominant genetic model [7,14,16,17,18,19,20].

Publication bias was assessed for both the −1195 G/A and −765 G/C polymorphisms using visual inspection of funnel plots and consideration of study distribution relative to expected symmetry under a fixed mean effect. The funnel plots were constructed with superimposed 95% confidence boundaries to facilitate detection of asymmetry or small-study effects (Figure 4).

For the −765 G/C polymorphism, the funnel plot appeared symmetrical, and all three included studies, including Prakash et al., fell within the expected 95% confidence triangle. This indicates a low likelihood of publication bias. Visual findings were consistent with the lack of heterogeneity among studies (I^2^ = 0%) and the robust overall effect estimate observed in the meta-analysis.

In the case of the −1195 G/A polymorphism, the funnel plot showed mild asymmetry. Most studies, including Prakash et al. [7], were correctly positioned within the confidence triangle after rechecking the odds ratio and standard error. However, one study—Dienha et al. [17]—remained a clear outlier, with a high odds ratio and wide confidence interval. This suggests the potential influence of a small-study effect or atypical population characteristics. Although Egger’s regression test was not formally conducted in this analysis, the asymmetry observed warrants cautious interpretation of the pooled effect size for this polymorphism.

## 4. Discussion

Cyclooxygenase-2 (COX-2) is a key inducible enzyme responsible for converting arachidonic acid into prostaglandins, particularly prostaglandin E_2_ (PGE_2_), a lipid involved in inflammation, vasodilation, and bone and soft tissue degradation in periodontal disease [21]. Given its pathophysiological relevance, genetic polymorphisms within the COX-2 gene have gained considerable attention as potential contributors to inter-individual variability in the presence of periodontal disease.

In this meta-analysis, we investigated the association of two promoter polymorphisms, −1195 G/A and −765 G/C, with the presence of periodontal disease. The present findings demonstrated a borderline significant association between the −1195 G/A variant and periodontal disease (OR = 1.86, 95% CI: 1.00–3.43, *p* = 0.05), while the −765 G/C polymorphism showed a statistically significant association (OR = 1.61, 95% CI: 1.12–2.32, *p* = 0.03). These findings suggest a stronger and more consistent association between the −765 G/C variant and periodontal disease. From the viewpoint of precision medicine, the identified genetic associations have meaningful clinical implications. Including COX-2 genotyping in risk assessments could support more personalized approaches to periodontal care. By identifying individuals with genetic predispositions, clinicians may be able to implement earlier preventive measures and consider tailored adjunctive treatments—such as COX-2 inhibitors—for those likely to exhibit a stronger inflammatory response. This strategy aligns with efforts to provide individualized, evidence-based care in periodontology. The observed strength of association, particularly for the −765 G/C polymorphism (OR = 1.61), suggests a moderate genetic effect size, consistent with multifactorial traits such as periodontal disease. The present findings also support the hypothesis that COX-2 promoter variants contribute to host susceptibility, likely by modulating gene expression and inflammatory responses.

The biological plausibility of these associations is grounded in the regulatory role of these polymorphisms in COX-2 gene expression. Variants such as −765 G/C have been associated with altered transcriptional activity, potentially leading to increased COX-2 expression and subsequent overproduction of PGE_2_ in inflamed periodontal tissues. This cascade may accelerate osteoclastic bone resorption and soft tissue degradation—hallmarks of periodontal disease pathogenesis.

The present study is consistent with prior case–control and meta-analytic studies. Li et al. [18] and Loo et al. [19], both included in our analysis, reported significant associations between the −765 C allele and elevated risk for periodontal disease in Chinese populations. Similarly, Zhang conducted a meta-analysis exclusively in Chinese individuals and concluded that the −765 G/C polymorphism was significantly increased with periodontal disease, particularly in studies using population-based controls. A subgroup analysis in that study found a strong association in the dominant model (GC + CC vs. GG, OR = 1.52, 95% CI: 1.24–1.86) and even more potent effects under recessive models (CC vs. GG + GC) [21].

Conversely, other studies have yielded conflicting results. For example, Fourmousis and Vlachos [22] reported a non-significant association in a mixed-ethnicity European cohort, underscoring the potential modifying effects of ethnicity and population structure. Moreover, Prakash et al. [7] reported non-significant associations in an Indian population, possibly reflecting differences in environmental exposures or gene–environment interactions.

Indeed, ethnicity appears to be a key moderator. In their meta-analysis, Jiang [12] noted that the A allele of −1195 G/A conferred increased periodontal disease risk in Asian populations, but not in Caucasians. Similarly, the results from Zhang et al. [21] showed opposite effects depending on the source of control, highlighting methodological factors that may drive inconsistency across studies.

Recent insights from genome-wide analyses and bioinformatics have further supported the polygenic nature of periodontal disease and its interaction with inflammatory pathways [23,24]. In particular, the identification of key immune-inflammatory genes, such as CXCL1, COL15A1, and CTSH, has broadened our understanding of host susceptibility beyond COX-2 polymorphisms. Loos et al. [24] emphasized that while individual polymorphisms may confer modest risk, the cumulative effect of multiple gene variants—particularly those involved in neutrophil recruitment and tissue remodeling—may substantially modulate disease expression. Additionally, Schaefer et al. [25] discussed how variations in genes encoding pattern-recognition receptors, cytokines, and eicosanoid-synthesizing enzymes (including COX-2) may interact with microbial challenges to drive disease heterogeneity. These findings are consistent with our conclusion that COX-2 variants, although relevant, likely represent only one aspect of a broader genetic predisposition to periodontal inflammation. The heterogeneity observed in our meta-analysis, particularly in studies using different diagnostic classifications or populations, may reflect such multifactorial influences.

Beyond genetic association analyses, the integration of diagnostic strategies such as saliva-based testing presents promising avenues for non-invasive, patient-friendly risk assessment in periodontology. Saliva provides a rich source of epithelial cells and microbial DNA, making it suitable not only for microbial diagnostics but also for host genomic analyses, including SNP detection. As demonstrated by Solomon et al. [26], saliva samples can yield PCR-amplifiable human genomic DNA under various storage conditions, using both commercial kits and cost-effective extraction protocols. These findings support the utility of saliva as a feasible biosample for large-scale, community-level genetic and periodontal screening. Future studies incorporating salivary biomarkers and genomic DNA may offer a more comprehensive and scalable diagnostic approach for periodontal diseases.

Despite the robustness of the present meta-analytic approach, several limitations must be acknowledged. First, variations in the definition and diagnosis of periodontal disease across included studies—ranging from the 1999 AAP classification to the 2017 AAP/EFP system—may have introduced clinical heterogeneity and affected comparability. Second, key confounding variables such as smoking status, diabetes, age, and gender were inconsistently reported or adjusted for, limiting the ability to control for their influence on genetic associations. Third, while the analysis focused on three well-studied COX-2 polymorphisms (−1195 G/A, −765 G/C, and 8473 T/C), data on other potentially relevant variants were lacking, thereby restricting the genetic scope of the review. Fourth, the moderate heterogeneity observed in the −1195 G/A analysis (I^2^ = 35%) may also reflect underlying differences in genetic background, diagnostic criteria, or environmental exposures. Importantly, studies from Caucasian populations were not represented, limiting the external validity of the findings. Fifth, several studies, including that of Dienha et al. [17], had small sample sizes, which may have led to imprecise estimates and wide confidence intervals. Finally, while publication bias appeared minimal for the −765 G/C polymorphism, mild asymmetry was observed in the funnel plot for the −1195 G/A polymorphism, suggesting possible small-study effects.

In comparison to previous meta-analyses, the present study offers notable advancements. For instance, Zhang et al. focused exclusively on Chinese populations and did not include the most recent studies from broader geographic regions, such as Iraq, India, and Ukraine [21]. In contrast, our analysis incorporated multiethnic cohorts, thus enhancing the generalizability of the findings beyond a single ethnic group. Furthermore, Jiang et al. [12] investigated multiple COX-2 SNPs but included fewer studies per variant and did not account for newer data published in the last decade.

Another important distinction lies in methodological rigor. Our meta-analysis adhered to the PRISMA 2020 guidelines, employing a comprehensive literature search strategy across six databases and applying strict inclusion criteria, excluding studies with inadequate control groups or violations of the Hardy–Weinberg equilibrium. Additionally, we conducted funnel plot analysis with 95% confidence boundaries and explored potential small-study effects that were not formally addressed in previous reports. This review brings several important updates to the current literature. To our knowledge, it is the first meta-analysis to comprehensively evaluate both the −765 G/C and the −1195 G/A polymorphisms of the *COX-2* gene in relation to chronic periodontitis. Earlier reviews have usually focused on a single variant or lacked a formal quantitative synthesis of the evidence. Moreover, our study incorporates more recent data, including several studies published after 2020, which were not included in previous analyses. By incorporating these newer findings, we aim to offer a more up-to-date and clinically relevant picture of the potential genetic links between *COX-2* variants and periodontal disease. Therefore, this study not only updates the evidence base but also builds on earlier analyses in terms of study selection, population diversity, and statistical assessment, offering a more robust and current evaluation of the role of COX-2 polymorphisms in periodontal disease.

From a clinical standpoint, the present findings suggest that COX-2 promoter polymorphisms, particularly the −765 G/C variant, may contribute to individual risk profiling in periodontal disease. As we move toward personalized periodontal care, genetic screening for high-risk alleles could inform preventive strategies and therapeutic decisions, especially in populations with a high prevalence of these variants. Anti-inflammatory therapies targeting COX-2 pathways may also have enhanced efficacy in genetically predisposed individuals. Future studies should prioritize multiethnic, well-powered, prospective cohorts with standardized diagnostic criteria and genotyping methods. Furthermore, mechanistic investigations linking COX-2 variants to expression levels and PGE_2_ production in periodontal tissues would deepen our understanding of gene function. Ultimately, studies examining gene–environment interactions, particularly in relation to tobacco use and microbiological profiles, are essential for elucidating the complex etiology of periodontal disease.

## 5. Conclusions

The findings of this systematic review and meta-analysis suggest that the COX-2 −1195 G/A polymorphism is significantly associated with increased susceptibility to periodontitis, particularly among Asian populations. In contrast, no significant association was observed for the −765 G/C polymorphism. The overall quality of the evidence ranged from low to moderate certainty, with heterogeneity and limited control for confounders in several studies. These results highlight a potential genetic influence in periodontal disease pathogenesis, specifically related to the COX-2 pathway.

## Figures and Tables

**Table 1:** Characteristics of included studies.

*Study*	Country	Ethnicity	Study Design	Definition of Periodontal Disease	Age (Mean or Range)	Gender (M/F)	Sample Size (Cases/Controls)	COX-2 Polymorphism(s)
*Daing et al. (2012) [16]*	India	South Asian	Case–control	Chronic periodontitis based on clinical and radiographic criteria	Not reported	Not reported	122/120	−1195 G/A
*Dahash et al. (2022) [14]*	Iraq	Middle Eastern	Case–control	Chronic periodontitis, clinical CAL, and PD criteria	22–55	45/25	70/30	−1195 G/A
*Dienha et al. (2023) [17]*	China	Han Chinese	Case–control	2017 AAP/EFP classification	56 (cases); 53 (controls)	26/30	56/20	−1195 G/A
*Li et al. (2012) [18]*	China	Han Chinese	Case–control	1999 AAP classification	54.4 ± 7.76 (cases); 36.9 ± 9.7 (controls)	115/7 (cases); 300/232 (controls)	122/53	−765 G/C
*Loo et al. (2011) [19]*	Malaysia	Southeast Asian	Case–control	Chronic periodontitis with CAL and PD criteria	21–50 (cases); 20–45 (controls)	66/80 (cases); 39/46 (controls)	146/85	−765 G/C
*Prakash et al. (2013) [7]*	India	South Asian	Case–control	Chronic periodontitis per 1999 classification	38.7 ± 7.5 (cases); 34.3 ± 8.2 (controls)	Not reported	200/200	−765 G/C, −1195 G/A
*Xie et al. (2009) [20]*	China	Han Chinese	Case–control	Periodontitis based on CAL and pocket depth	Mean age not provided; range: 21–52	Not reported	146/108	−1195 G/A

**Table 2:** Newcastle–Ottawa Scale (NOS) assessment of included studies.

Study	Case Def.	Case Rep.	Control Sel.	Control Def.	Confounding	Exposure Asc.	Same Method	Non-Resp.	Total Score	Quality
**Daing et al. (2012) [16]**	1	0	0	1	1	1	1	0	5	Low
**Dahash et al. (2022) [14]**	1	0	0	1	1	1	1	0	5	Low
**Dienha et al. (2023) [17]**	1	0	0	0	0	1	1	1	4	Low
**Li et al. (2012) [18]**	1	0	1	1	2	1	1	0	7	High
**Loo et al. (2011) [19]**	1	0	1	1	2	1	1	0	7	High
**Prakash et al. (2013) [7]**	1	0	0	1	2	1	1	0	5	Low
**Xie et al. (2009) [20]**	1	0	0	1	2	1	1	0	6	Moderate

Case Def.: case definition. Case Rep.: representativeness of cases. Control Sel.: selection of controls. Control Def.: definition of controls. Confounding: control for confounders. Exposure Asc.: ascertainment of exposure. Same Method: the same ascertainment method was used for cases and controls. Non-Resp.: non-response rate. Quality was assessed using the Newcastle–Ottawa Scale (NOS), with scores ranging from 0 to 9. Studies scoring 7–9 were considered of high quality, those scoring 4–6 as moderate quality, and those with 0–3 as low quality. The total score reflects the cumulative points awarded across three domains: selection (4 points), comparability (2 points), and exposure (3 points).

**Table 3:** Genotypic distributions and effect estimates from individual studies (dominant model).

Study	Polymorphism	Genotypes (Cases)	Genotypes (Controls)	Dominant Model Comparison	OR (95% CI)	*p*-Value
**Daing et al. (2012) [16]**	−765 G/C	GG: 55, GC: 33, CC: 34	GG: 316, GC: 204, CC: 12	GC + CC vs. GG	2.59 (1.93–3.47)	0.000
**Dahash et al. (2022) [14]**	−765 G/C	GG: 134, GC: 68, CC: 78	GG: 152, GC: 90, CC: 8	GC + CC vs. GG	2.48 (1.89–3.26)	0.0001
**Dienha et al. (2023) [17]**	−765 G/C	GG: 133, GC: 58, CC: 9	GG: 145, GC: 47, CC: 8	GC + CC vs. GG	1.42 (0.90–2.24)	0.132
**Li et al. (2012) [18]**	−1195 G/A	GG: 19, GA: 77, AA: 50	GG: 40, GA: 64, AA: 44	GA + AA vs. GG	2.49 (1.33–4.69)	0.033
**Loo et al. (2011) [19]**	−1195 G/A	GG: 2, GA: 8, AA: 12	GG: 0, GA: 1, AA: 11	GA + AA vs. GG	6.00 (1.05–71.08)	0.024
**Prakash et al. (2013) [7]**	−1195 G/A	GG: 10, GA: 27, AA: 19	GG: 20, GA: 25, AA: 15	GA + AA vs. GG	1.41 (0.89–2.21)	—
**Xie et al. (2009) [20]**	−1195 G/A	GG: 147, GA: 46, AA: 7	GG: 154, GA: 42, AA: 4	GA + AA vs. GG	1.14 (0.71–1.84)	0.578

Note: Dominant model compares (GA + AA) vs. GG for −1195 G/A, and (GC + CC) vs. GG for −765 G/C.

## Data Availability

All data analyzed in this study were extracted from previously published studies cited in the manuscript. No new data were generated. The search strategy and full dataset are available upon reasonable request from the corresponding author.

## Appendix A

**PRISMA 2020 checklist for systematic review manuscript.**

**Table:**

No.	PRISMA Item	Description	Reported on Page/Section
1	Title	Identify the report as a systematic review.	Title page
2	Abstract	Structured summary per PRISMA guidelines.	Abstract
3	Rationale	Describe the rationale for the review.	Introduction, paragraphs 1–3
4	Objectives	Provide an explicit statement of the objective(s).	End of introduction
5	Eligibility criteria	Specify inclusion/exclusion criteria and how studies were grouped.	Methods: Eligibility Criteria
6	Information sources	Describe all sources (databases, registers, etc.) used.	Methods: Information Sources
7	Search strategy	Present full search strategy for all databases.	Suppl. Appendix
8	Selection process	Specify methods for study selection.	Methods: Study Selection
9	Data collection process	Specify methods for data extraction.	Methods: Data Extraction
10a	Data items (Outcomes)	Define all outcomes for which data were sought.	Methods: Data Extraction; Results Table 3
10b	Data items (Other)	Report other variables (e.g., population characteristics).	Methods: Data Extraction; Table 1
11	Study risk of bias assessment	Specify methods for risk of bias assessment.	Methods: Quality Assessment; Table 2
12	Effect measures	Specify effect measures used (e.g., ORs, CIs).	Methods: Statistical Analysis
13a	Synthesis methods	Describe criteria for synthesis eligibility.	Methods: Statistical Analysis
13b	Preparation of data	Methods for tabulation or transformation.	Methods: Data Extraction
13c	Methods to explore heterogeneity	Describe methods (e.g., subgroup analysis).	Methods: Statistical Analysis
13d	Sensitivity analysis	Describe methods to assess robustness.	Methods: Sensitivity Analysis (implicit)
14	Reporting bias assessment	Methods for assessing risk of reporting bias.	Methods: Publication Bias
15	Certainty assessment	Describe any methods to assess certainty in the evidence.	Results
16a	Study selection	Describe number of studies screened, included, with reasons.	Results: Study Selection, Figure 1
16b	Excluded studies	Cite and explain excluded studies.	Results: Study Selection
17	Study characteristics	Present characteristics for each study.	Results: Table 1
18	Risk of bias in studies	Present risk of bias for each study.	Results: Table 2
19	Results of individual studies	Provide summary statistics and effect estimates.	Results: Table 3
20a	Results of syntheses	Summarize synthesized findings.	Results: Synthesis of Results
20b	Heterogeneity	Report measures of heterogeneity (I^2^).	Results: Synthesis of Results
20c	Subgroup/sensitivity analyses	Report results of sensitivity analysis.	Results: Discussion (brief discussion)
21	Reporting biases	Present results of reporting bias assessments.	Results: Publication Bias
22	Certainty of evidence	Present certainty in evidence.	Results, Appendix
23a	Interpretation	Interpret results in context of other evidence.	Discussion
23b	Limitations of evidence	Discuss limitations of included studies.	Discussion
23c	Limitations of review process	Discuss limitations of the review methods.	Discussion
23d	Implications	Discuss implications for practice and future research.	Discussion and Conclusion
24a	Registration	Provide registry name and registration number.	Methods: Protocol (PROSPERO: CRD420251008939)
24b	Protocol access	Indicate where protocol can be accessed.	Methods: Link included
25	Support	Describe funding or support for the review.	Author Contributions/No funding declared
26	Competing interests	Declare any competing interests.	Conflicts of Interest
27	Availability of data	Report availability of data, code, and materials.	Data Availability Statement

**PRISMA 2020 search strategy reporting template.**

**Table:**

Section	Item	Reported Detail
Information sources	Databases and platforms used	PubMed (MEDLINE), Scopus, Web of Science, EMBASE (via Ovid), Cochrane Library; supplementary sources: Google Scholar, manual reference screening
	Dates of search	Initial search conducted from 11 to 15 March 2025; supplementary screening (gray literature and reference lists) from 17 to 20 March 2025
	Timeframe covered	From database inception to 20 March 2025
	Restrictions applied	Included only peer-reviewed, full-text articles published in English; excluded animal studies, in vitro studies, reviews, editorials, conference abstracts, and studies lacking genotype data
Search strategy	Search terms and Boolean logic	(“COX-2” OR “cyclooxygenase-2”) AND (“polymorphism” OR “SNP” OR “single nucleotide polymorphism”) AND (“periodontitis” OR “periodontal disease”)
Controlled vocabulary terms	MeSH terms (PubMed), Emtree (EMBASE) were used where applicable	
	Syntax adapted to database	Yes—syntax adjusted to database-specific conventions (e.g., TITLE-ABS-KEY for Scopus, TS for Web of Science)
Filters applied	Language: English; publication type: Original research articles; population: humans	

**Detailed search strategy by database (as per PRISMA 2020).**

**Table:**

Database	Search Strategy
PubMed	((“Chronic Periodontitis”[MeSH Terms]) OR “Chronic Periodontitis”[Title/Abstract] OR “Chronic Periodontitides”[Title/Abstract] OR “Periodontitides, Chronic”[Title/Abstract] OR “Periodontitis, Chronic”[Title/Abstract] OR “Adult Periodontitis”[Title/Abstract] OR “Adult Periodontitides”[Title/Abstract] OR “Periodontitides, Adult”[Title/Abstract] OR “Periodontitis, Adult”[Title/Abstract] OR “Periodontal disease”[Title/Abstract] OR “Periodontal treatment”[Title/Abstract] OR “Periodontal therapy”[Title/Abstract]) AND ((“Cyclooxygenase 2”[MeSH Terms]) OR “COX-2”[Title/Abstract] OR “Cyclooxygenase-2”[Title/Abstract] OR “Cyclooxygenase 2”[Title/Abstract]) AND (“Polymorphism, Single Nucleotide”[MeSH Terms] OR “SNP”[Title/Abstract] OR “single nucleotide polymorphism”[Title/Abstract] OR “genetic variant”[Title/Abstract] OR “genetic polymorphism”[Title/Abstract])
Scopus	(TITLE-ABS-KEY(“chronic periodonti*”) OR TITLE-ABS-KEY(“periodontitides, chronic”) OR TITLE-ABS-KEY(“periodontitis, chronic”) OR TITLE-ABS-KEY(“adult periodonti*”) OR TITLE-ABS-KEY(“periodontitides, adult”) OR TITLE-ABS-KEY(“periodontitis, adult”) OR TITLE-ABS-KEY(“periodontal disease”) OR TITLE-ABS-KEY(“periodontal therapy”) OR TITLE-ABS-KEY(“periodontal treatment”)) AND (TITLE-ABS-KEY(“COX-2”) OR TITLE-ABS-KEY(“cyclooxygenase-2”) OR TITLE-ABS-KEY(“cyclooxygenase 2”)) AND (TITLE-ABS-KEY(“SNP”) OR TITLE-ABS-KEY(“single nucleotide polymorphism”) OR TITLE-ABS-KEY(“genetic variant”) OR TITLE-ABS-KEY(“genetic polymorphism”))
EMBASE (Ovid)	(‘chronic periodontitis’/exp OR ‘chronic periodontitis’:ti,ab OR ‘chronic periodontitides’:ti,ab OR ‘adult periodontitis’:ti,ab OR ‘periodontitis, adult’:ti,ab OR ‘periodontal disease’:ti,ab OR ‘periodontal therapy’:ti,ab OR ‘periodontal treatment’:ti,ab) AND (‘cyclooxygenase 2′/exp OR ‘COX-2′:ti,ab OR ‘cyclooxygenase-2’:ti,ab OR ‘cyclooxygenase 2’:ti,ab) AND (‘single nucleotide polymorphism’/exp OR ‘SNP’:ti,ab OR ‘single nucleotide polymorphism’:ti,ab OR ‘genetic polymorphism’:ti,ab OR ‘genetic variant’:ti,ab)
Web of Science	(TS = (“chronic periodontitis”) OR TS = (“chronic periodontitides”) OR TS = (“adult periodontitis”) OR TS = (“periodontitis, chronic”) OR TS = (“periodontitis, adult”) OR TS = (“periodontal therapy”) OR TS = (“periodontal disease”) OR TS = (“periodontal treatment”)) AND (TS = (“COX-2”) OR TS = (“cyclooxygenase-2”) OR TS = (“cyclooxygenase 2”)) AND (TS = (“SNP”) OR TS = (“single nucleotide polymorphism”) OR TS = (“genetic polymorphism”) OR TS = (“genetic variant”))
Cochrane Library	(“chronic periodontitis” OR “chronic periodontitides” OR “adult periodontitis” OR “periodontal disease” OR “periodontal therapy” OR “periodontal treatment”) AND (“COX-2” OR “cyclooxygenase-2” OR “cyclooxygenase 2”) AND (“SNP” OR “single nucleotide polymorphism” OR “genetic polymorphism” OR “genetic variant”)

**GRADE assessment summary table.**

**Table:**

Outcome	Study Design	Risk of Bias	Inconsistency	Indirectness	Imprecision	Publication Bias	Other Factors	Certainty of Evidence
−765 G/C (rs20417)	Observational	Serious (some low-quality studies)	Not serious (I^2^ = 0%)	Not serious	Not serious (narrow CI)	None detected	Large effect (OR = 1.61)	Moderate
−1195 G/A (rs689466)	Observational	Serious (4/7 studies low quality)	Some concern (I^2^ = 35%)	Not serious	Serious (borderline CI: 1.00–3.43; includes null)	Possible (funnel plot asymmetry)	None	Low
